# The Poplar MYB Master Switches Bind to the SMRE Site and Activate the Secondary Wall Biosynthetic Program during Wood Formation

**DOI:** 10.1371/journal.pone.0069219

**Published:** 2013-07-29

**Authors:** Ruiqin Zhong, Ryan L. McCarthy, Marziyeh Haghighat, Zheng-Hua Ye

**Affiliations:** Department of Plant Biology, University of Georgia, Athens, Georgia, United States of America; University of Massachusetts Amherst, United States of America

## Abstract

Wood is mainly composed of secondary walls, which constitute the most abundant stored carbon produced by vascular plants. Understanding the molecular mechanisms controlling secondary wall deposition during wood formation is not only an important issue in plant biology but also critical for providing molecular tools to custom-design wood composition suited for diverse end uses. Past molecular and genetic studies have revealed a transcriptional network encompassing a group of wood-associated NAC and MYB transcription factors that are involved in the regulation of the secondary wall biosynthetic program during wood formation in poplar trees. Here, we report the functional characterization of poplar orthologs of MYB46 and MYB83 that are known to be master switches of secondary wall biosynthesis in Arabidopsis. In addition to the two previously-described PtrMYB3 and PtrMYB20, two other MYBs, PtrMYB2 and PtrMYB21, were shown to be MYB46/MYB83 orthologs by complementation and overexpression studies in Arabidopsis. The functional roles of these PtrMYBs in regulating secondary wall biosynthesis were further demonstrated in transgenic poplar plants showing an ectopic deposition of secondary walls in PtrMYB overexpressors and a reduction of secondary wall thickening in their dominant repressors. Furthermore, PtrMYB2/3/20/21 together with two other tree MYBs, the *Eucalyptus* EgMYB2 and the pine PtMYB4, were shown to differentially bind to and activate the eight variants of the 7-bp SMRE consensus sequence, composed of ACC(A/T)A(A/C)(T/C). Together, our results indicate that the tree MYBs, PtrMYB2/3/20/21, EgMYB2 and PtMYB4, are master transcriptional switches that activate the SMRE sites in the promoters of target genes and thereby regulate secondary wall biosynthesis during wood formation.

## Introduction

Wood is produced by the activity of the vascular cambium, and it encompasses a complex developmental program involving the differentiation of the vascular cambium into secondary xylem mother cells, cell elongation, secondary wall deposition, programmed cell death, and finally heartwood formation [1]. Genomic studies of wood formation have revealed thousands of genes that are induced during wood formation [2]–[7], some of which are transcriptional regulators suggested to be involved in the regulation of various developmental steps of wood development, such as cambial activity and secondary xylem differentiation [8]–[11]. Since wood is not only an important raw material for many industrial applications, such as building construction, pulping and paper making, and furniture, but also considered to be a promising source for biofuel production [12], uncovering the molecular switches controlling various steps of wood development could potentially provide strategies for custom designing of wood components tailored for diverse end needs.

Wood at maturity is essentially the remains of secondary walls largely composed of cellulose, hemicelluloses and lignin. Therefore, understanding how the biosynthesis of secondary wall components is regulated could potentially provide genetic tools for altering wood composition. Since many genes are required for the biosynthesis of each major wood component, it is conceivable that the biosynthetic genes responsible for the making of wood components are coordinately activated during wood development. Recent molecular and genetic studies in tree species have demonstrated that the coordinated activation of wood biosynthetic genes is mediated by a transcriptional network involving multileveled transcriptional controls. It was found that a group of wood-associated NAC domain transcription factors (WNDs) are the top master switches regulating the expression of a number of downstream transcription factors, which ultimately lead to the biosynthesis of secondary walls during wood formation in tree species [13]–[16], [17]. These tree NAC master switches are functional orthologs of secondary wall NAC master switches (SWNs) found in a number of non-woody species, such as Arabidopsis, rice, maize, *Brachypodium*, and alfalfa [13], [18]–[21], suggesting the evolutionary conservation of SWNs in regulating secondary wall biosynthesis. A number of PtrWND-induced downstream transcription factors, including PtrMYB3, PtrMYB20, PtrMYB18, PtrMYB74, PtrMYB75, PtrMYB121, PtrMYB128, PtrZF1, PtrGATA1, PtrNAC150, PtrNAC156, and PtrNAC157, have been shown to activate the promoters of genes involved in the biosynthesis of cellulose, xylan and lignin [15], indicating that the transcriptional control of secondary wall biosynthesis in tree species is a complex process involving multiple levels of regulation.

The poplar MYB genes, *PtrMYB3* and *PtrMYB20*, together with two other MYB genes, *EgMYB2* [22] and *PtMYB4* [23], from *Eucalyptus* and pine, respectively, have previously been shown to be functional orthologs of the Arabidopsis *MYB46* and *MYB83* [13], [24], indicating the evolutionary conservation of MYB46 and its close homologs in the regulation of secondary wall biosynthesis in vascular plants. MYB46 and MYB83 have been demonstrated to bind to the 7-bp secondary wall MYB-responsive element (SMRE), ACC(A/T)A(A/C)(T/C), and directly activate a suite of transcription factors and secondary wall biosynthetic genes [25]. MYB46 was shown in another study to bind to the M46R motif [26], which is the same as the SMRE sequence except for one additional nucleotide that is included. The SMRE consensus sequence encompasses eight variants of SMRE sequences. These SMRE sequences include three AC element sequences, AC-I, AC-II and AC-III, which were previously shown to be the cis-elements involved in the regulation of expression of lignin biosynthetic genes [27], [28]. Although early reports suggest that EgMYB2 and PtMYB4 regulate lignin biosynthesis via activating the AC elements present in the promoters of lignin biosynthetic genes [22], [23], it remains to be investigated whether the tree orthologs of MYB46 and MYB83, such as PtrMYB3, PtrMYB20, EgMYB2, and PtMYB4, behave as MYB46 and MYB83 in the binding of the SMRE sequences. Furthermore, the functional roles of these tree MYBs in wood formation have not been examined in tree species.

In this report, we investigated the effects of overexpression and dominant repression of poplar orthologs of MYB46 and MYB83 on wood formation in poplar trees, and determined the cis-elements these tree MYBs bind. We show that beside the previously described PtrMYB3 and PtrMYB20, two additional MYBs, PtrMYB2 and PtrMYB21, are functional orthologs of MYB46 and MYB83, capable of activating the biosynthetic genes for cellulose, xylan and lignin. Overexpression and dominant repression of these PtrMYB in transgenic poplar lead to an ectopic deposition of secondary walls and a reduction in secondary wall thickening, respectively. Furthermore, we demonstrate that the tree MYBs, including PtrMYB2/3/20/21, EgMYB2 and PtMYB4, all bind to and activate the SMRE sequences. Our findings indicate that MYB46 and its orthologs in both herbaceous Arabidopsis and tree species activate their downstream target genes via binding and activating the SMRE sites.

## Results

### PtrMYB2 and PtrMYB21 are able to complement the Arabidopsis *myb46 myb83* double mutant

**Figure 1:**
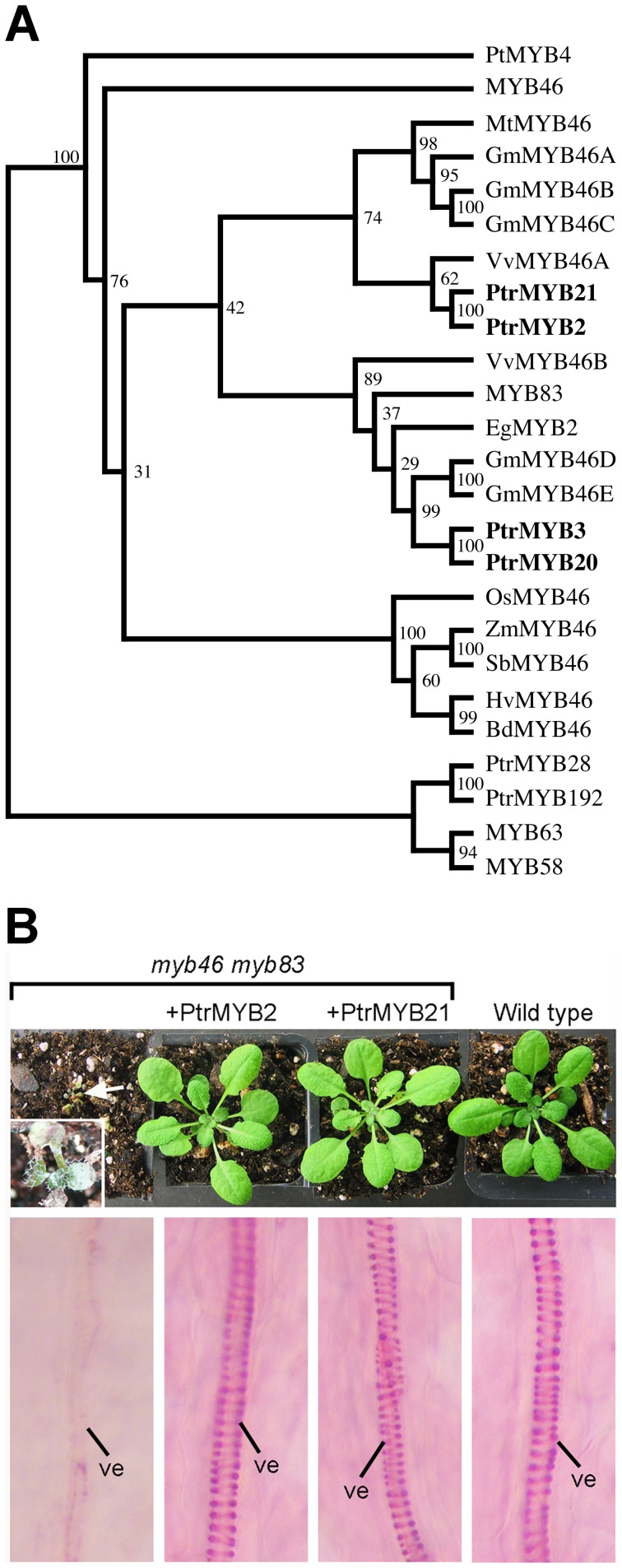
PtrMYB2 and PtrMYB21 are able to functionally complement the growth arrest and vessel wall-thickening defects in the Arabidopsis *myb46 myb83* double mutant. (A) Phylogenetic relationship of Arabidopsis MYB46/MYB83 and their orthologs from poplar (*Populus trichocarpa*; PtrMYB2/3/20/21) and other plants, including *Eucalyptus* (*Eucalyptus grandis*; EgMYB2); Pine (*Pinus taeda*; PtMYB4), grapevine (*Vitis vinifera*; VvMYB46), alfalfa (*Medicago truncatula*; MtMYB46), soybean (*Glycine max*; GmMYB46), rice (*Oryza sativa*; OsMYB46), maize (*Zea mays*; ZmMYB46), sorghum (*Sorghum bicolor*; SbMYB46), barley (*Hordeum vulgare*; HvMYB46), and brachypodium (*Brachypodium distachyon*; BdMYB46). The phylogenetic tree was constructed with the neighbor-joining algorithm using PHYLIP and displayed using the TREEVIEW program. Bootstrap values are shown in percentages at the nodes. MYB58, MYB63 and their poplar homologs (PtrMYB28 and PtrMYB192) are included as the outgroup. (B) Complementation of *myb46 myb83* by PtrMYB2 and PtrMYB21. Upper panel shows four-week-old seedlings of the Arabidopsis *myb46 myb83* double mutant (arrow; higher magnification of *myb46 myb83* in inset), the *myb46 myb83* mutant expressing PtrMYB2 (+PtrMYB2), the *myb46 myb83* mutant expressing PtrMYB21 (+PtrMYB21), and the wild type. The lower panel shows secondary wall thickening in leaf veins of corresponding plants displayed above. Note that the vein in the *myb46 myb83* mutant has little secondary wall thickening, which is rescued by the expression of PtrMYB2 or PtrMYB21.

Our previous study of PtrWND functions showed that the expression of *PtrMYB2* and *PtrMYB21* is induced by PtrWNDs, indicating that PtrMYB2 and PtrMYB21 are PtrWND-regulated downstream transcription factors involved in transcriptional regulation of wood formation [15]. Phylogenetic analysis revealed that *PtrMYB2* and *PtrMYB2*1 are grouped together with the Arabidopsis *MYB46* and *MYB83* genes, two other poplar MYB genes (*PtrMYB3* and *PtrMYB20*) that were previously shown to be functional orthologs of MYB46 [24], and many additional MYB46 homologs from other plant species (Fig. 1A). To elucidate the functional roles of PtrMYB2 and PtrMYB21, we first tested their ability to rescue the mutant phenotypes conferred by the Arabidopsis *myb46 myb83* double mutant. The *myb46 myb83* mutant exhibited a strongly retarded seedling growth phenotype and a defect in secondary wall thickening in leaf vessels (Fig. 1B) [29]. Expression of either PtrMYB2 or PtrMYB21 completely restored the normal plant development and the normal secondary wall thickening in leaf vessels in the *myb46 myb83* mutant. These results indicate that together with PtrMYB3 and PtrMYB20 [24], PtrMYB2 and PtrMYB21 are Arabidopsis MYB46 and MYB83 functional orthologs involved in the regulation of secondary wall biosynthesis.

### Overexpression of PtrMYB2 and PtrMYB21 in Arabidopsis results in activation of the entire secondary wall biosynthetic program

**Figure 2:**
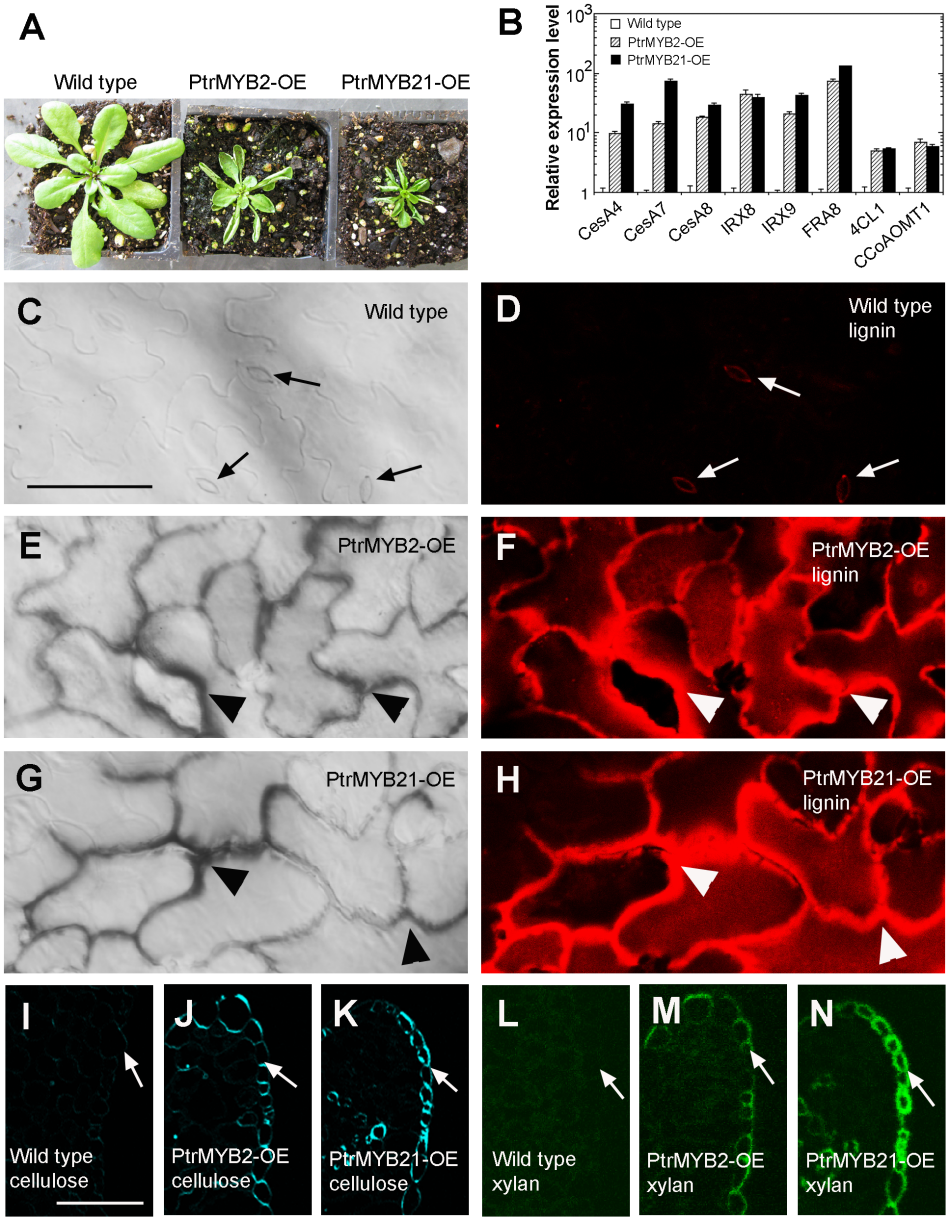
Overexpression of PtrMYB2 and PtrMYB21 in Arabidopsis induces ectopic deposition of secondary walls in leaf epidermal cells. Three-week-old transgenic plants expressing *PtrMYB2* or *PtrMYB21* driven by the CaMV 35S promoter was examined for induction of secondary wall biosynthetic genes and ectopic deposition of secondary wall components. Bar in (C)  = 64 µm for (C) to (H) and bar in (I)  = 60 µm for (I) to (N). (A) Three-day-old seedlings of the wild type (left), a PtrMYB2 overexpressor (PtrMYB2-OE; middle), and a PtrMYB21 overexpressor (PtrMYB21-OE; right). Note the upward curly leaves in PtrMYB2-OE and PtrMYB21-OE. (B) Quantitative PCR analysis showing the induction of expression of secondary wall biosynthetic genes for cellulose (*CesA4*, *CesA7* and *CesA8*), xylan (*FRA8*, *IRX8* and *IRX9*) and lignin (*4CL1* and *CCoAOMT1*). The expression level of genes of interest in the wild type is set to 1. Error bars denote SE of three biological replicates. (C) and (D) Differential interference contrast (DIC) image (C) and lignin autofluorescence image (D) of the epidermis of a wild-type leaf. Note the low lignin signal in the inner wall of guard cells (arrows). (E) and (F) DIC image (E) and lignin autofluorescence image (F) of the leaf epidermis of a PtrMYB2 overexpressor showing ectopic wall thickening and lignin signal (arrowheads), respectively. (G) and (H) DIC image (G) and lignin autofluorescence image (H) of the leaf epidermis of a PtrMYB21 overexpressor showing ectopic wall thickening and lignin signal (arrowheads), respectively. (I) to (K) Sections of leaves of the wild type (I), PtrMYB2-OE (J) and PtrMYB21-OE (K) stained for cellulose with Calcofluor White. Note the strong signal for cellulose staining in the epidermal walls (arrows) of PtrMYB2-OE and PtrMYB21-OE. (L) to (N) Sections of leaves of the wild type (L), PtrMYB2-OE (M) and PtrMYB21-OE (N) stained for xylan with the LM10 xylan antibody. Note the strong signal for xylan staining in the epidermal walls (arrows) of PtrMYB2-OE and PtrMYB21-OE.

**Figure 3:**
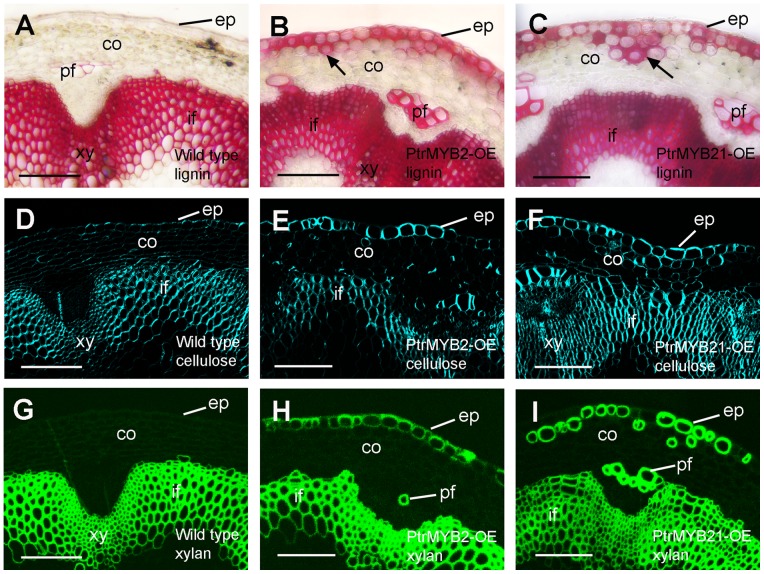
Induction of ectopic deposition of secondary wall components in the epidermis of Arabidopsis stems by overexpression of PtrMYB2 and PtrMYB21. Cross sections of stems of the wild type (A, D, G), PtrMYB2-OE (B, E, H) and PtrMYB21-OE (C, F, I) were stained for lignin with phloroglucinol (A to C), cellulose with Calcofluor White (D to F) and xylan with the LM10 xylan antibody (G to I). Note the ectopic deposition of lignin, cellulose and xylan in the epidermis and some cortical cells (arrows) of PtrMYB2-OE and PtrMYB21-OE. co, cortex; ep, epidermis; if, interfascicular fiber; pf, phloem fiber; xy, xylem. bars  = 67 µm.

The finding that PtrMYB2 and PtrMYB21 are able to complement the *myb46 myb83* mutant phenotypes prompted us to investigate whether they are capable of activating the entire secondary wall biosynthetic program when overexpressed in Arabidopsis. Examination of the PtrMYB overexpressors revealed that their leaves were curled upward (Fig. 2A), which resembles the typical MYB46-induced leaf curling phenotype due to the ectopic deposition of secondary walls in the epidermal cells in the upper side of leaves [30]. Indeed, the epidermal cell walls in the upper side of leaves in both PtrMYB2 and PtrMYB21 overexpressors were significantly thickened (Fig. 2E, G) with an ectopic deposition of lignin (Fig. 2F, H). In contrast, the epidermal cell walls in the upper side of wild-type leaves were thin (Fig. 2C) and lacked lignin signals except for the guard cells in which the ventral walls exhibited low lignin signals (Fig. 2D). In addition to lignin, cellulose (Fig. 2J, K) and xylan (Fig. 2M, N) were ectopically deposited in the epidermal cell walls in the upper side of leaves in both PtrMYB2 and PtrMYB21 overexpressors, indicating that overexpression of PtrMYB2 and PtrMYB21 leads to an ectopic deposition of secondary wall components. The induction of ectopic secondary wall deposition by PtrMYB2 and PtrMYB21 was also observed in stems in which lignin, cellulose and xylan were heavily laid in the walls of epidermal cells and some cortical cells (Fig. 3). Quantitative PCR analysis further confirmed that overexpression of PtrMYB2 and PtrMYB21 resulted in a drastic induction in the expression of secondary wall biosynthetic genes, including *CesA4*, *CesA7* and *CesA8* for cellulose, *IRX8*, *IRX9* and *FRA8* for xylan, and *4CL1* and *CCoAOMT1* for lignin (Fig. 2B). Together, these results indicate that like PtrMYB3 and PtrMYB20 (24), PtrMYB2 and PtrMYB21 are transcriptional regulators controlling secondary wall biosynthesis.

### Regulation of secondary wall biosynthesis during wood formation by PtrMYB2/3/20/21 genes in poplar trees

**Figure 4:**
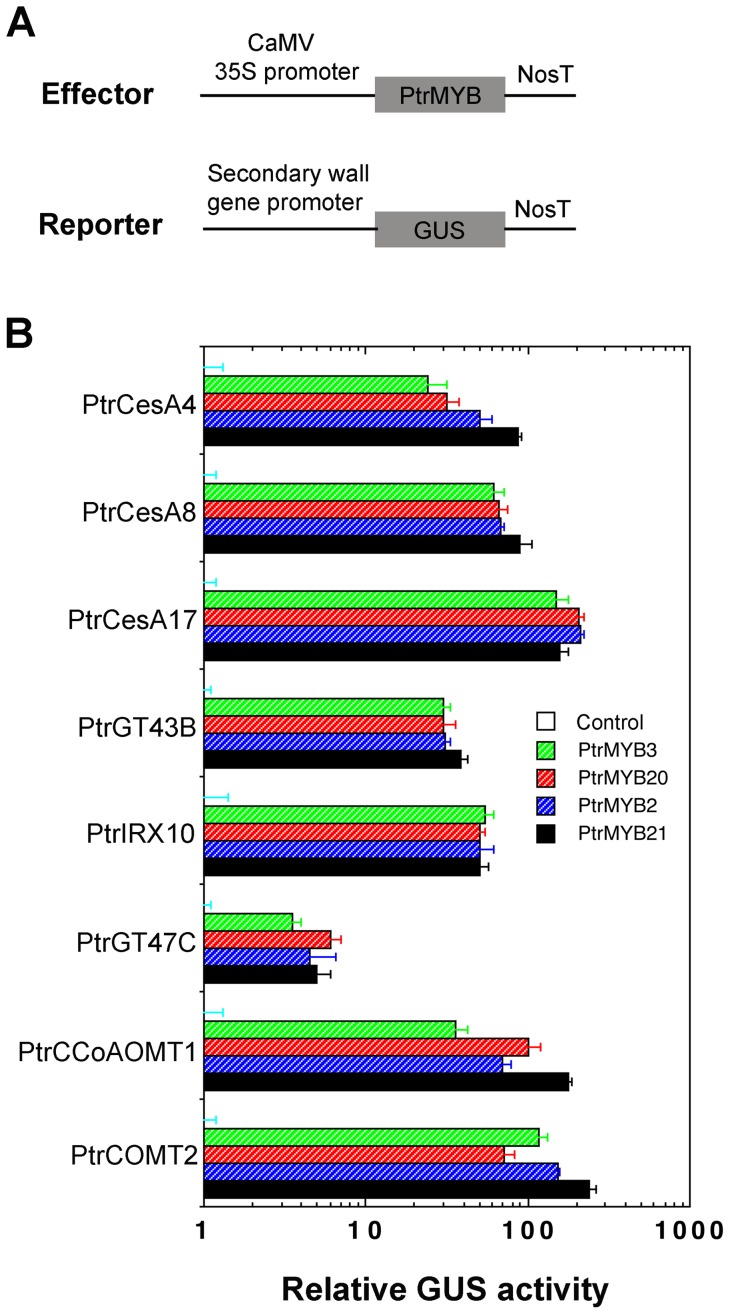
The promoters of poplar secondary wall biosynthetic genes are activated by poplar MYB master switches. (A) Diagrams of the effector and reporter constructs used for the transactivation analysis. NosT, nopaline synthase terminator. (B) Transactivation analysis showing the activation by poplar MYB master switches of the GUS reporter gene driven by various secondary wall biosynthetic gene promoters. The effector and reporter constructs were cotransfected into Arabidopsis leaf protoplasts and after incubation, the transfected protoplasts were used for GUS activity assay. The GUS activity in protoplasts transfected with the reporter construct and an effector construct without MYB genes was set to 1. Error bars denote the SE of three biological replicates.

**Figure 5:**
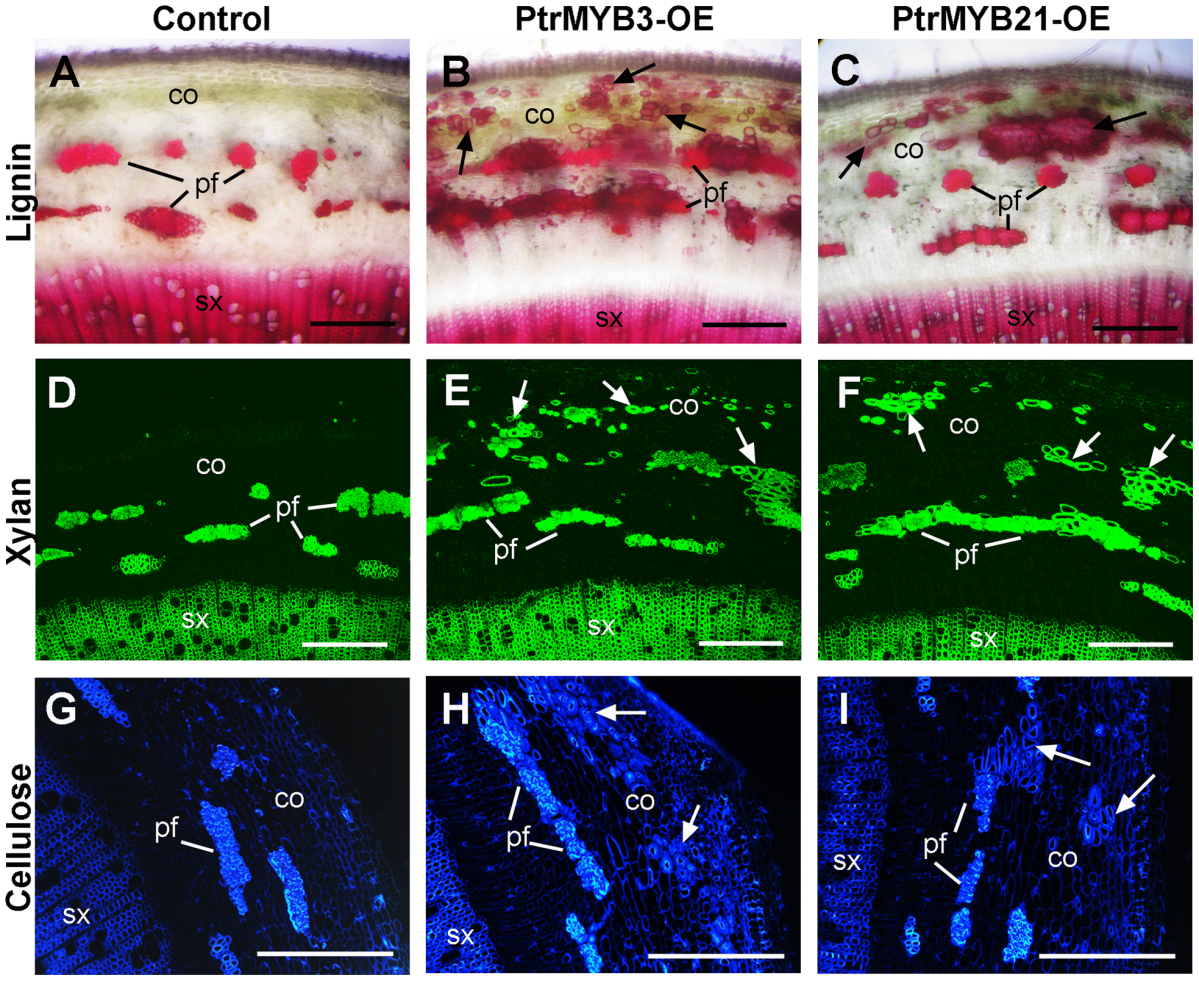
Overexpression of PtrMYB3 and PtrMYB21 causes ectopic deposition of secondary wall components in transgenic poplar stems. Stems of the transgenic control (transformed with the empty vector only), PtrMYB3-OE and PtrMYB21-OE were sectioned and stained for lignin with phloroglucinol-HCl, xylan with the LM10 xylan antibody, and cellulose with Calcofluor White. (A) to (C) Lignin staining of stem sections showing ectopic lignin deposition in cortical cells (arrows) in PtrMYB3-OE (B) and PtrMYB21-OE (C) compared with the control (A). (D) to (F) Xylan staining of stem sections showing ectopic xylan deposition in cortical cells (arrows) in PtrMYB3-OE (E) and PtrMYB21-OE (F) compared with the control (D). (G) to (I) Cellulose staining of stem sections showing ectopic cellulose deposition in cortical cells (arrows) in PtrMYB3-OE (H) and PtrMYB21-OE (I) compared with the control (G). co, cortex; pf, phloem fiber; sx, secondary xylem. Bars  = 228 µm.

We next extended our investigation of the functions of PtrMYB2, PtrMYB3, PtrMYB20 and PtrMYB21 in poplar trees using both overexpression and dominant repression approaches. We first tested whether these PtrMYBs were able to activate the promoters of poplar secondary wall biosynthetic genes using transactivation assay in Arabidopsis leaf protoplasts. It was found that all four MYBs were able to activate the expression of the GUS reporter gene driven by the promoters of the poplar secondary wall biosynthetic genes for cellulose, xylan and lignin (Fig. 4), demonstrating that these MYB genes are transcriptional regulators of secondary wall biosynthesis in poplar trees. We next examined the effects of overexpression of these PtrMYBs in poplar (*Populus alba* X *Populus tremula*). Because *PtrMYB3* and *PtrMYB20* are duplicated genes and so are *PtrMYB2* and *PtrMYB21* [5], we chose *PtrMYB3* and *PtrMYB21*, one from each pair of the duplicated genes for further analysis. Transgenic poplar MYB overexpressors had shorter stems with smaller leaves compared with the control ones transformed with the empty vector. Staining of stem sections for lignin, xylan and cellulose revealed that in addition to the normal staining of secondary wall components in secondary xylem and phloem fiber cells as seen in the control (Fig. 5A, D, G), the walls of some of cortical cells in the PtrMYB overexpressors were stained intensively for lignin (Fig. 5B, C), xylan (Fig. 5E, F), and cellulose (Fig. 5H, I). The results from transactivation and overexpression analyses demonstrate that these PtrMYBs are capable of activating the secondary wall biosynthetic program in poplar trees, leading to the deposition of secondary wall components, including cellulose, xylan and lignin.

**Figure 6:**
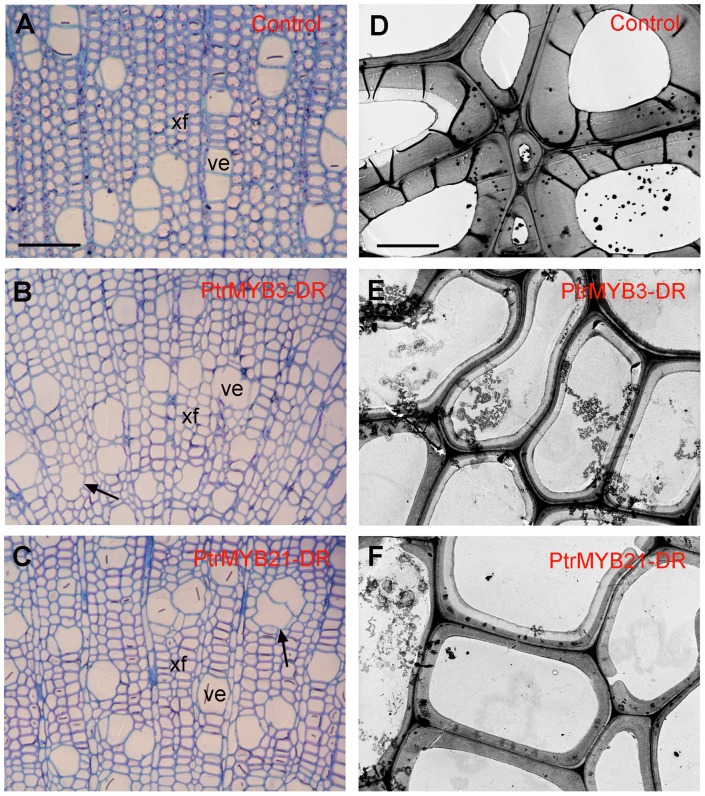
Reduction in secondary wall thickening and alteration in vessel morphology in the wood of transgenic poplar with dominant repression of PtrMYB3 (PtrMYB3-DR) and PtrMYB21 (PtrMYB21-DR). The bottom parts of 6-month-old transgenic poplar plants were sectioned for examination of wood anatomy. The control is transgenic plants transformed with the empty vector only. (A) to (C) Toluidine blue-stained wood sections showing thinner secondary walls in xylary fibers and deformed vessel morphology in PtrMYB3-DR (B) and PtrMYB21-DR (C) compared with the control (A). (D) to (F) Transmission electron microscopy of wood sections showing reduced wall thickness in xylary fibers in PtrMYB3-DR (E) and PtrMYB21-DR (F) compared with the control (D). ve, vessel; xf, xylary fiber. Bar in (A)  = 94 µm for (A) to (C), and bar in (D)  = 4.9 µm for (D) to (F).

The functional roles of these PtrMYBs in wood formation were further examined by repression of their functions in poplar. Considering that the four PtrMYBs are close homologs activating secondary wall biosynthesis, we chose to use the EAR-dominant repression approach [31] to overcome their functional redundancy. Similar to the overexpression study, *PtrMYB3* and *PtrMYB21* were chosen for construction of the chimeric gene containing PtrMYB fused with the EAR repression domain at the C-terminus. Examination of transgenic poplar trees (*Populus alba* X *Populus tremula*) expressing the dominant repressors showed a reduced stem height and strong alterations in wall thickness and vessel morphology in the wood of stems (Fig. 6). Compared to the control transformed with the empty vector (Fig. 6A, D), the thickness of secondary walls in the wood fibers in both PtrMYB3 and PtrMYB21 repressors was reduced by 60 to 70% (Fig. 6B, C, E, F). Furthermore, some vessels were apparently deformed in their morphology in the PtrMYB repressors compared with the control, a common phenotype observed in transgenic poplar trees with reduced secondary wall thickening [15], [32]. Together, the results from the overexepression and dominant repression studies provide direct evidence demonstrating that these PtrMYBs are transcriptional switches controlling secondary wall biosynthesis during wood formation in poplar trees.

### Analysis of cis-element sequences activated by PtrMYB2/3/20/21

**Figure 7:**
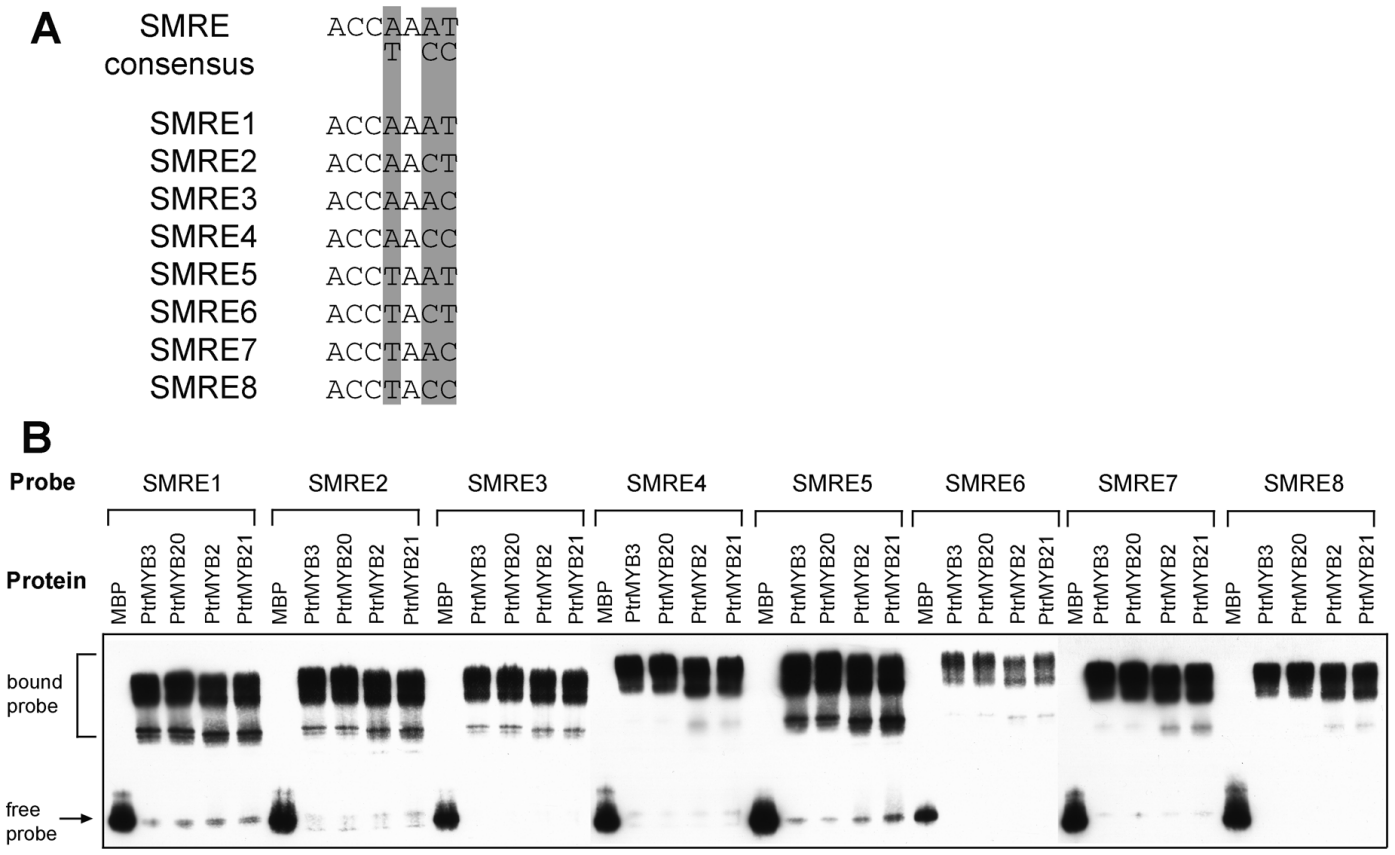
EMSA of binding of PtrMYB3, PtrMYB20, PtrMYB2, and PtrMYB21 to the SMRE sequences. (A) Shown are the SMRE consensus sequence and eight SMRE variants. (B) EMSA showing that PtrMYB3, PtrMYB20, PtrMYB2, and PtrMYB21 all bind to the eight SMRE sequences. MBP, maltose binding protein. Each biotin-labeled SMRE probe was incubated with fusion proteins and the bound probes were separated from the free ones, which were detected by the chemiluminescent method.

To investigate how these PtrMYBs activate the secondary wall biosynthetic program, we set out to determine the cis-element sequences that they bind to. Since the four PtrMYBs have been shown to be able to complement the Arabidopsis *myb46 myb83* double mutant phenotypes (Fig. 1B) [24], they very likely bind to and activate the same cis-elements as those of MYB46 and MYB83, thus leading to activation of secondary wall biosynthesis. MYB46 and MYB83 have previously been demonstrated to bind to the secondary wall MYB-responsive element (SMRE) composed of a 7-bp consensus sequence, ACC(A/T)A(A/C)(A/T) (Fig. 7A) [25]. To test whether these PtrMYBs also bind to the SMRE sites, we employed the electrophoretic mobility shift assay (EMSA) to investigate their ability of binding various SMRE sequences. It was found that incubation of the recombinant PtrMYBs, including PtrMYB3, PtrMYB20, PtrMYB2 and PtrMYB21, with biotin-labeled SMRE sequences resulted in a shift of the SMRE probes (Fig. 7B). It was evident that these PtrMYBs effectively bound to all the eight variants of SMRE sequences, demonstrating that these PtrMYBs bind to the same SMRE consensus sequence as MYB46/MYB83. It was noted that PtrMYBs led to multiple band shifts of the SMRE probes, which was likely resulted from binding of the probes by the monomeric and dimeric forms of MYB proteins. The differences in the band shift pattern among different SMRE probes were likely due to the differential affinity of binding of PtrMYBs to various SMRE sequences.

**Figure 8:**
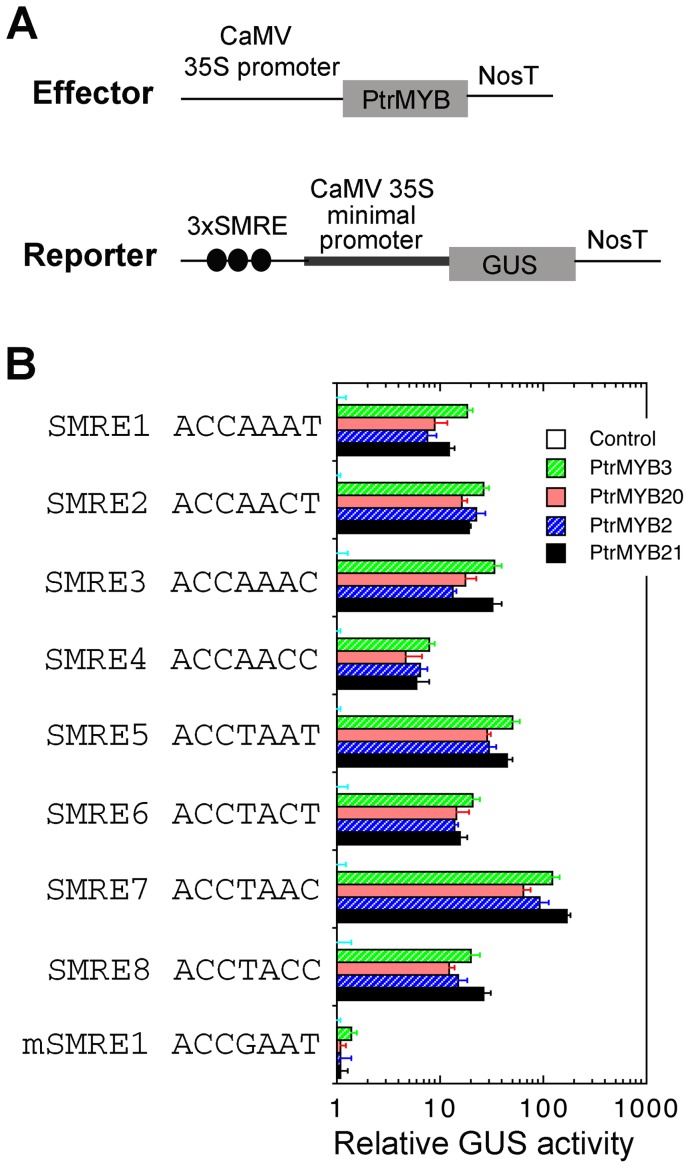
Activation of SMRE-driven GUS reporter gene expression by PtrMYBs. (A) Diagrams of the effector and reporter constructs used for the transactivation analysis. 3xSMRE, three copies of the SMRE sequence. (B) Transactivation analysis showing that PtrMYBs effectively activated the expression of the SMRE-driven GUS reporter gene. The reporter and effector constructs (A) were co-transfected into Arabidopsis leaf protoplasts and after incubation, the transfected protoplasts were lysed and analyzed for the GUS activity. The control is the GUS activity in protoplasts transfected with the reporter construct and an empty effector construct without PtrMYBs and taken as 1. Error bars are the SE of three biological replicates.

We next applied the transactivation analysis to test the ability of these PtrMYBs in activation of the SMRE sites in vivo. The PtrMYB effector construct and the SMRE-driven GUS reporter construct were co-transfected into Arabidopsis leaf protoplasts (Fig. 8A). Analysis of the GUS activity revealed that these PtrMYBs effectively activated all eight variants of SMRE sites (Fig. 8B) but not the mutated SMRE site (mSMRE1) that has a substitution of the conserved fourth nucleotide (A/T) with a G. It was also noted that the activation strength by these PtrMYBs varied significantly among different SMRE sequences. For example, the activation of the SMRE7 sequence was ten-fold higher than that of the SMRE4 sequence. These results indicate that these PtrMYBs activate their target gene expression via binding to the SMRE sites albeit at different binding affinities among the eight variants of the SMRE sequences.

### Binding and activation of the SMRE sites by EgMYB2 and PtMYB4

**Figure 9:**
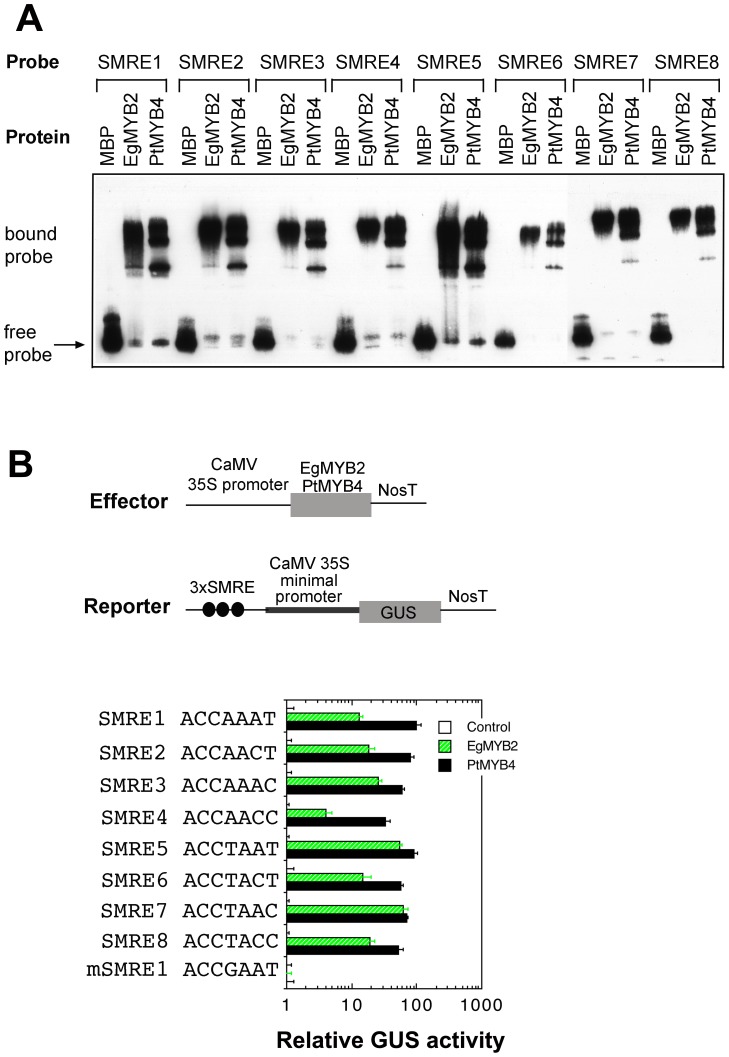
EgMYB2 and PtMYB4 bind to and activate the SMRE sequences. (A) EMSA showing the binding of the eight SMRE sequences by EgMYB2 and PtMYB4. MBP and fusion proteins (EgMYB2 and PtMYB4) were incubated with biotin-labeled SMRE probes and the bound probes were separated from the free ones, which were detected by the chemiluminescent method. (B) Transactivation analysis showing the activation of the SMRE-driven GUS reporter gene by EgMYB2 and PtMYB4 (lower panel). The reporter and effector constructs (upper panel) were co-transfected into Arabidopsis leaf protoplasts and after incubation, the transfected protoplasts were lysed and analyzed for the GUS activity. The control is the GUS activity in protoplasts transfected with the reporter construct and an empty effector construct without EgMYB2 or PtMYB4 and taken as 1. Error bars are the SE of three biological replicates.

In addition to PtrMYB2/3/20/21, two other tree MYBs, EgMYB2 and PtMYB4 from *Eucalyptus* and pine, respectively, are also functional orthologs of MYB46/MYB83 [13]. To further substantiate this finding, we investigated whether EgMYB2 and PtMYB4 bind to and activate the same cis-element as these PtrMYBs. EMSA study demonstrated that both EgMYB2 and PtMYB4 were able to cause a mobility shift of all eight variants of the SMRE probes, indicating that they are effective in binding to the SMRE sequences (Fig. 9A). The differences in the band shift pattern between EgMYB2 and PtMYB4 were likely due to the differential affinity of binding of these MYBs to the SMRE sequences. Transactivation analysis showed that EgMYB2 and PtMYB4 were able to activate the GUS reporter gene driven by all eight variants of the SMRE sequences but not the mutated mSMRE1 sequence (Fig. 9B). The activation strength by EgMYB2 varied significantly among the eight variants of SMREs, indicating differential affinity of EgMYB2 binding to various SMRE sequences. It appeared that PtMYB4 exhibited a higher binding affinity to SMREs than EgMYB2, as the activation of SMRE-driven GUS expression by PtrMYB4 was generally higher than EgMYB2 (Fig. 9B). Together, these results indicate that like MYB46/MYB83 and PtrMYB2/3/20/21, EgMYB2 and PtMYB4 bind to the SMRE sites to activate their target genes, thus leading to activation of secondary wall biosynthesis.

## Discussion

### Four poplar MYBs function as second-level master switches regulating wood formation

Several lines of molecular and genetic evidence demonstrate that four homologous poplar MYB transcription factors, PtrMYB2/3/20/21, are transcriptional master switches controlling secondary wall biosynthesis during wood formation. First, expression of these *PtrMYBs* is able to rescue the secondary wall defects conferred by double mutations of the Arabidopsis *MYB46* and *MYB83* genes. Second, when overexpressed in Arabidopsis and poplar trees, they are capable of activating the secondary wall biosynthetic genes, leading to ectopic deposition of secondary walls. Third, dominant repression of these PtrMYB functions in poplar trees results in a defect in secondary wall thickening in wood. The findings that these four PtrMYBs are functional orthologs of the Arabidopsis MYB46/MYB83 and they all are capable of activating secondary wall biosynthetic genes in poplar trees indicate that these PtrMYBs might function redundantly in regulating secondary wall biosynthesis during wood formation. One intriguing question is why poplar evolved to retain all these four PtrMYBs. One possibility is that although they are all transcriptional activators of secondary wall biosynthesis, they may play differential roles in different organs and/or cell types, which is consistent with previous transcriptome analysis showing that they exhibit differential expression patterns in different organs and tissues [5]. Another possibility is that they might differentially activate their target genes as they show differential binding affinity toward different SMRE sequences that are present in promoters of their target genes (see discussion below). Therefore, the expression of these four PtrMYBs might be required for a full strength of transcriptional activation of secondary wall biosynthesis. In Arabidopsis, MYB46 and MYB83 appear to function redundantly in the activation of secondary wall biosynthesis as T-DNA knockout mutation of either MYB46 or MYB83 alone does not cause an apparent reduction in secondary wall thickening [29].

**Figure 10:**
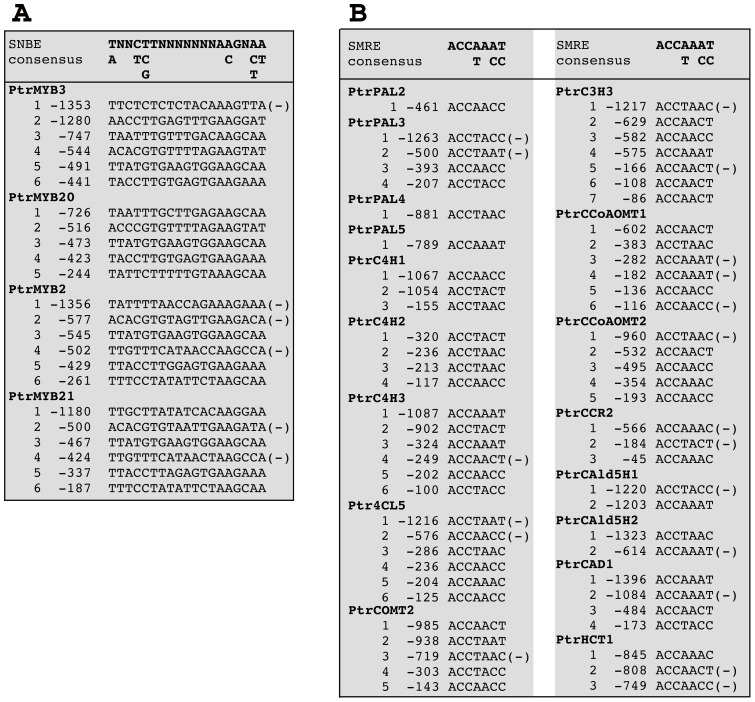
Identification of the SNBE sequences in the 1.5-kb promoters of PtrMYB2/3/20/21 genes based on the SNBE consensus sequence (A) and the SMRE sequences in the 1.5-kb promoters of poplar lignin biosynthetic genes based on the SMRE consensus sequence (B). The number shown at the left of each sequence denotes the position of the first nucleotide relative to the start codon. The plus or minus symbol at the right indicates the sequence from the forward or reverse strand of DNA, respectively.

A previous study on poplar PtrWNDs has shown that the expression of all these four *PtrMYBs* is induced by PtrWNDs [15]. Like the Arabidopsis SWNs [33], PtrWNDs bind to the SNBE sites in the promoters of their direct target genes and thereby activate their expression [15]. Examination of the promoter sequences of the four PtrMYBs revealed the presence of multiple SNBE sites (Fig. 10A). Two of them, PtrMYB3 and PtrMYB20, have previously been demonstrated to be direct targets of PtrWNDs [24], and the SNBE sites from the promoters of PtrMYB3 and PtrMYB21 have been shown to be bound and activated by PtrWNDs [15]. These findings indicate that these four PtrMYBs are direct targets of PtrWNDs and they act as second-level master switches in the PtrWND-mediated transcriptional network regulating secondary wall biosynthesis during wood formation.

### PtrMYB2/3/20/21 bind to and activate the SMRE sequences

Both EMSA and transactivation analyses demonstrated that like the Arabidopsis MYB46 and MYB83, the four PtrMYBs are able to bind to the eight variants of the SMRE sequences, indicating that they activate their target gene expression through binding to the SMRE sites in the promoters of their target genes. The conservation of the DNA binding sites between these PtrMYBs and MYB46/MYB83 provides the molecular basis for the ability of these PtrMYBs to complement the *myb46 myb83* mutant phenotypes. It is interesting to note that the four PtrMYBs exhibit a large variation of binding affinity among the SMRE sequences with the highest affinity toward SMRE7 (Fig. 8), implying that the activation strength of their direct target genes by these PtrMYBs may depend on the particular SMRE sequences present in the promoters of their direct targets.

We further demonstrated that EgMYB2 and PtMYB4, orthologs of MYB46/MYB83 from *Eucalyptus* and pine, respectively, also bind to and activate the SMRE sequences. Early work showed that EgMYB2 and PtMYB4 bind to and activate the AC elements, including AC-I, ACII and AC-III, and they were thought to specifically regulate lignin biosynthesis through activating the AC elements [22], [23]. Our work extends these early findings by showing that these tree MYBs activate not only the three AC elements that are identical to three SMRE variants (SMRE8/AC-I; SMRE4/AC-II; and SMRE7/AC-III) but also five additional variants of the SMRE sequences. The ability of these tree MYBs to bind to the AC elements as well as other SMRE sequences indicates that they are not specific to regulating lignin biosynthetic genes, which is consistent with the findings that these tree MYBs are master switches capable of activating the biosynthetic pathways for not only lignin but also cellulose and xylan [13].

**Figure 11:**
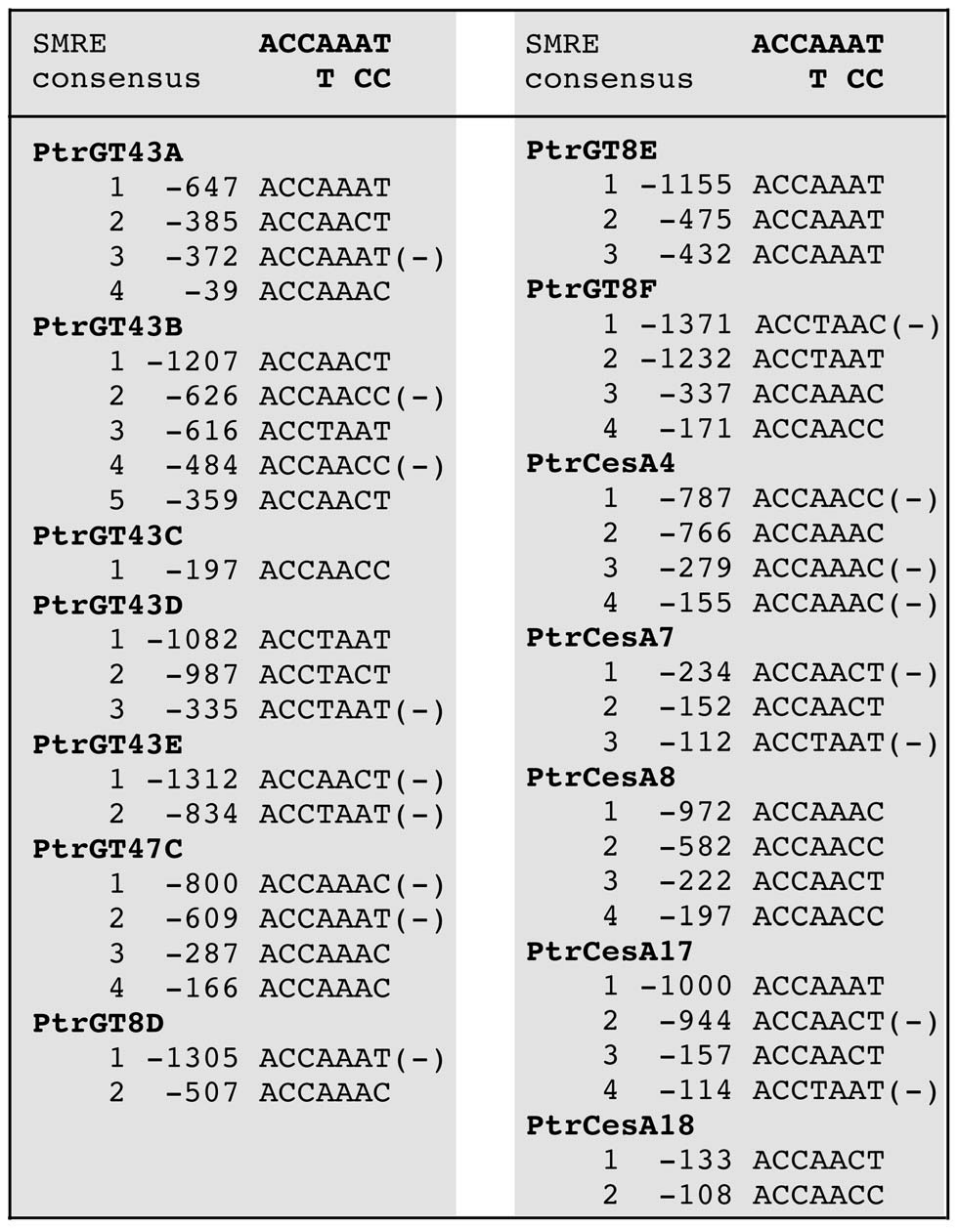
Identification of the SNBE sequences in the 1.5-kb promoters of of poplar cellulose and xylan biosynthetic genes based on the SMRE consensus sequence.

It has long been thought that lignin-specific MYBs bind to the AC elements in the promoters of lignin biosynthetic genes and thereby activate the lignin biosynthetic pathway [27], [28]. In Arabidopsis, two MYB genes, *MYB58* and *MYB63*, have been shown to bind to the AC elements and regulate the biosynthetic genes for lignin but not cellulose and xylan, which is congruent with the proposed mode of regulation of lignin gene expression via the AC cis-elements [34]. However, the finding that the Arabidopsis MYB46 and MYB83 also activate lignin biosynthetic genes via binding to the AC elements and other SMRE sites indicate that the regulation of lignin biosynthesis is much more complicated than previously thought. Examination of 18 poplar core lignin biosynthetic genes [35] revealed that in addition to the AC elements, other five variants of the SMRE sites are also present in the promoters of these genes (Fig. 10B). These findings support the hypothesis that the expression of lignin biosynthetic genes is regulated by the coordinated actions of multiple MYBs, including activators and repressors [22], [23], [25], [34], [36]–[40], via binding to not only the AC elements but also other SMRE sites. In addition to the promoters of lignin biosynthetic genes, those of cellulose and xylan biosynthetic genes also contain multiple SMRE sequences (Fig. 11), indicating that the PtrMYBs could potentially bind to and activate the SMRE sites in the promoters of cellulose and xylan biosynthetic genes. However, direct activation analysis with Arabidopsis MYB46 demonstrated that it directly activated the expression of a few xylan biosynthetic genes but did not directly induce the expression of cellulose biosynthetic genes [25], suggesting that MYB46 alone is not sufficient to directly activate the expression of cellulose biosynthetic genes. It is likely that these MYB master switches function cooperatively with other secondary wall-associated transcription factors in activating their target genes.

### Evolutionary conservation of MYB46/MYB83 and their orthologs in regulating secondary wall biosynthesis

Close homologs of MYB46 and MYB83 exist in the genomes of both gymnosperms and angiosperms that have available genome sequences or expressed sequence tags (Fig. 1A). The homologs from pine, *Eucalyptus*, poplar, rice and maize have all been demonstrated to be functional orthologs of MYB46 and MYB83 and are capable of activating the biosynthetic pathways for cellulose, xylan and lignin [13], [15], [24]. In Arabidopsis and poplar trees, these MYBs function as second-level master switches in the SWNs/WNDs-mediated transcriptional network controlling secondary wall biosynthesis, indicating the evolutionary conservation of the transcriptional regulatory networks controlling secondary wall biosynthesis. Further identification and functional characterization of transcriptional regulators controlling secondary wall biosynthesis in poplar trees will contribute to our understanding of how wood formation is regulated at the molecular level in tree species, the knowledge of which could be applied to genetically improve wood composition for diverse end uses.

## Methods

### Phylogenetic analysis

The phylogenetic tree was constructed by first aligning the MYB protein sequences with the ClustalW2 program [41] and then analyzing their relationship with the neighbor-joining algorithm using the Phylogeny Inference Package (PHYLIP) [42]. The tree was displayed using the TREEVIEW program [43]. The statistical significance of the tree was tested by bootstrap analysis using 1000 bootstrap trials in the PHYLIP program.

### Complementation of Arabidopsis *myb46 myb83* mutant

The full-length cDNAs of *PtrMYB2* (5′-atgaggaagccagaggcctctgg-3′ and 5′-tcaactttggaaatcaagagaaggaca-3′) and *PtrMYB21* (5′-atgaggaagccagaggcctct-3′ and 5′-tcattggaaatcaaggaatggaaaggc-3′) driven by the 3-kb *MYB46* promoter were cloned into the pGPTV vector and introduced into the *myb46* (−/−; homozygous) *myb83* (+/−; heterozygous) double mutant [29]. More than 60 transgenic plants were generated for each construct and screened for double homozygous *myb46 (−/−) myb83 (−/−)* mutants by PCR amplification of T-DNA insertions as described previously [29]. Among the complemented plants, about 70% showed normal growth as the wild type, and the rest of them were partially rescued with a slower growth and smaller leaves compared with the wild type. At least 10 complemented *myb46 myb83* double mutant plants that showed normal growth as the wild type were chosen for examination of plant growth and vessel morphology phenotypes.

### Overexpression and dominant repression of PtrMYBs

The PtrMYB overexpression constructs (PtrMYB2-OE, PtrMYB21-OE, and PtrMYB3-OE) were created by ligating the full-length *PtrMYB2*, *PtrMYB21*, or *PtrMYB3* (5′-atgaggaagccggatctaatg-3′ and 5′-tgtaagtgtagcctgttataaaacttgg-3′) cDNA downstream of the CaMV 35S promoter in pBI121. The PtrMYB dominant repression constructs (PtrMYB3-DR and PtrMYB21-DR) were generated by fusing the full-length *PtrMYB3* or *PtrMYB21* cDNA in frame with the dominant EAR repression sequence [31], which was ligated downstream of the CaMV 35S promoter in pBI121. The PtrMYB2-OE and PtrMYB21-OE constructs were introduced into wild-type *Arabidopsis thaliana* (ecotype Columbia) by agrobacterium-mediated transformation. For each construct, at least 60 transgenic Arabidopsis plants were generated for phenotypic analyses. The overexpression and dominant repression constructs were also introduced into poplar trees (*Populus alba* x *tremula*) by agrobacterium-mediated transformation as described [44]. The transgenic poplar seedlings were selected and grown in a greenhouse. Transgenic poplar plants transformed with an empty vector were used as the control. For each construct, at least 20 independent transgenic poplar lines were confirmed by PCR for the presence of the transgene in the genome and used for morphological and histological analyses.

### Histology

For light microscopy, stem segments embedded in low viscosity (Spurr's) resin (Electron Microscopy Sciences) were cut into 1-µm-thick sections with a microtome and stained with toluidine blue [45]. For transmission electron microscopy, 85-nm-thick sections were cut, poststained with uranyl acetate and lead citrate, and observed using a Zeiss EM 902A transmission electron microscope (Carl Zeiss). Presence of lignin was visualized by staining the sections with phloroglucinol-HCl or using a UV fluorescence microscope [46]. Secondary wall cellulose staining was done by incubating 1-µm-thick sections with 0.01% Calcofluor White [47]. Under the conditions used, only secondary walls exhibited brilliant fluorescence. Xylan was detected by using the monoclonal LM10 antibody against xylan and fluorescein isothiocyanate-conjugated goat anti-rat secondary antibodies [48].

### Gene Expression Analysis

Total RNA was isolated from leaves with a Qiagen RNA isolation kit (Qiagen). First strand cDNAs were synthesized from the total RNA treated with DNase I and then used as a template for PCR analysis. The real-time quantitative PCR was performed with the QuantiTect SYBR Green PCR kit (Clontech) using first strand cDNAs as templates. The relative expression level of each gene was calculated by normalizing the PCR threshold cycle number of each gene with that of the *EF1α* reference gene in each sample. The efficiencies of the PCR reactions among the samples were compared and found to be very similar between the genes analyzed and the *EF1α* reference gene, which range from 78% to 80%. The data were the average of three biological replicates.

### Transactivation Analysis

To test the ability of tree MYBs to activate poplar secondary wall biosynthetic gene promoters [15], the reporter construct containing the GUS reporter gene driven by a 2-kb promoter of the poplar gene of interest and the effector construct containing MYBs driven by the CaMV 35S promoter were co-transfected into Arabidopsis leaf protoplasts [49]. To test the ability of MYBs to activate the SMRE sites, the reporter construct containing the GUS reporter gene driven by three copies of various SMRE sequences and the effector construct containing MYBs driven by the CaMV 35S promoter were co-transfected into Arabidopsis leaf protoplasts. Another construct containing the firefly luciferase gene driven by the CaMV 35S promoter was included in each transfection for determination of the transfection efficiency. After 20-hr incubation, protoplasts were lysed and the supernatants were subjected to assay of the GUS and luciferase activities [50]. The GUS activity was normalized against the luciferase activity in each transfection, and the data are the average of three biological replicates.

### Electrophoretic Mobility Shift Assay

The tree MYBs were fused in frame with the maltose-binding protein (MBP) and expressed in *Escherichia coli*. The recombinant MYB-MBP protein was purified using amylose resin and then used for electrophoretic mobility shift assay (EMSA). The biotin-labeled SMRE oligonucleotides were incubated with 100 ng of MYB-MBP in the binding buffer [10 mM Tris, pH 7.5, 50 mM KCl, 1 mM DTT, 2.5% glycerol, 5 mM MgCl_2_, 0.05% Nonidet P-40, and 100 ng/mL poly(dI-dC)]. The MYB-bound DNA probes were separated from the unbound ones by polyacrylamide gel electrophoresis, electroblotted onto nitrocellulose membrane and detected by the chemiluminescent method [30].

### Statistical Analysis

The experimental data of the quantitative PCR analysis and GUS activity assay were subjected to statistical analysis using the Student's *t* test program (http://www.graphpad.com/quickcalcs/ttest1.cfm), and the quantitative difference between the two groups of data for comparison in each experiment was found to be statistically significant (p<0.001).

### Accession numbers

The GenBank accession numbers for the genes used in this study are PtrMYB3 (KF148675), PtrMYB20 (KF148676), PtrMYB2 (KF148677), PtrMYB21 (KF148678), MYB46 (At5g12870), MYB83 (At3g08500), EgMYB2 (AJ576023), PtMYB4 (AY356371), MYB58 (At1g16490), MYB63 (At1g79180), OsMYB46 (JN634084), ZmMYB46 (JN634085), SbMYB46 (XP_002443268), HvMYB46 (AAU43823), BdMYB46 (XP_003575963), VvMYB46A (XP_002282821), VvMYB46B (XP_002275467), MtMYB46 (XP_003597423), GmMYB46A (XP_003542045), GmMYB46B (XP_003539482), GmMYB46C (XP_003539066), GmMYB46D (XP_003543900), GmMYB46E (XP_003555035), PtrMYB28 (XM_002307154), PtrMYB192 (XM_002310643), CesA4 (At5g44030), CesA7 (At5g17420), CesA8 (At4g18780), FRA8 (At2g28110), IRX8 (At5g54690), IRX9 (At2g37090), 4CL1 (At1g51680), CCoAOMT1 (At4g34050), PtrCesA4 (XM_002301820), PtrCesA7 (XM_002308376), PtrCesA8 (XM_002316779), PtrCesA17 (XM_002325086), PtrCesA18 (XM_002305024), PtrGT43A (JF518934), PtrGT43B (JF518935), PtrGT43C (JF518936), PtrGT43D (JF518937), PtrGT43E (JF518938), PtrGT47C (XM_002314266), PtrGT8D (XM_002319766), PtrGT8E (XM_002310744), PtrGT8F (XM_002326863), PtrCCoAOMT1 (XM_002313089), and PtrCOMT2 (XM_002317802).
